# Metropolitan Tuberculosis Difficulties

**Published:** 1919-05-10

**Authors:** 


### METROPOLITAN TUBERCULOSIS DIFFICULTIES.

The shortcomings in the public treatment of tuberculosis in London are further illustrated in a report to be submitted by a sanatorium benefit sub-committee to the Insurance Committee for the County of London. The President of the Local Government Board and the Chairman of the National Health Insurance Joint Committee were asked for assistance in drawing up a comprehensive scheme of action which has notoriously long been wanting, especially as, in addition, points of difference have arisen between the L.C.C. and the Insurance Committee in relation to such important matters as the standard of accommodation to be adopted, and the minimum number of beds. The report relates evidence that further efforts to arrive at an understanding with the Council on these heads have been fruitless. Of the two officials mentioned, the first-named states that he is prepared to ask the Council to guarantee 100 more beds for insured persons than the funds of the Committee would provide, if this would bring the two bodies together. The latter, reminding all parties that the Sanatorium Benefit Sub-Committee is budgeting this year for a deficiency of approximately £40,000, suggests in effect adjudication on the dispute by the Ministry of Health. . . . A further report states that the Sanatorium Benefit Sub-Committee is of opinion that the Downs Sanatorium, which was built as a fever block for children, is radically unsuitable for the treatment of tuberculosis in grown men; that this unsuitability (especially the comparative smallness of ground) largely accounts for the "spirit of unrest" amongst the patients which was manifested in the complaints that led to the Sub-Committee's visit; and that the Metropolitan Asylums Board should be asked to expedite the provision of sanatoria specially adapted for the treatment of tuberculous male patients. This painful tale, which has now been familiar for years past, has a good many causes. To begin with, there is the mode of passing of the original Insurance Act. That measure was rushed through without sufficient elaboration, partly to gain kudos for its promoters, partly to try to keep pace with Germany. Then there are the confused strata of Metropolitan municipal administration—a mighty maze, and quite without a plan, it might almost be said, even since the emergence of the L.C.C. These political points we do not touch, save to suggest two things: The first, that the passion for domestic legislative enactment which is subsequently quite disregarded (*e.g.*, against black smoke from factory chimneys, successful in Belgium or Germany, uniformly transgressed here, or against "cut-outs" in motor-cycles) is profitless; the second, that the Ministry of Health may be wished a happy issue out of all these troubles. On the more professional aspects of the topic under notice we may, however, be more explicit, taking the last point first. It has apparently taken this Insurance Committee six years (1913-1919) to find out that a fever block for children does not make a good sanatorium for adults; and, by implication, that a body dealing with juvenile zymotic disease and quite without experience of tuberculosis, but with a reputation for a meticulous administrative routine, was not a good body to go to for the care of all the curable consumptives in London. Well, we could have told the Insurance Committee for the County of London this before; did so tell them, in fact, twice: Once, when the M.A.B., being given charge of this new work, proceeded to appoint as sanatorium superintendents not people with tuberculosis experience, but their own officers, smallpox experts, and so forth; and again when the result happened which we had predicted, a "row," complaints, and an official inquiry. This incident, it seems now, has been repeated at least once. Such incidents as these, which we hasten to say are not confined to London, show the real need there is for a centralised tuberculosis administration with a proper staff of experts. We could suggest four good names as a nucleus, Drs. Gauvain, Latham, Morland, and Rivière. It is highly important to have no "freak" appointments to such a staff, however strongly suggested by suffrage and other lay societies, for it is probably fear of such which is leading local administrative bodies to begin a rather natural, but mischievous, quiet fortifying against that central medical directive authority which would make for purity and efficiency of administration, and which in the case under notice—the case of tuberculosis—is especially recommended by so liberal and popular a mind as Professor Sir William Osler's. And we have spoken plainly on this affair, because plain speaking in the Press is the great want of the time. The laity is seeing for itself, however, that even the small privations we have had in England have multiplied tuberculosis alarmingly. It is drawing the correct conclusion—that the previous medical teaching that infection was practically the only thing that mattered from the point of view of prophylaxis, was quite wrong. The munition girls did not run into infection in their factories. But they felt the constitutional strain of industrialism just as men had done before the war, and therefore their death-rate from tubercle rose. So far, the only acknowledgment of this error we have seen outside our own columns was in a certain leading article in *The Lancet* which caused a rare flutter in orthodox northern dovecots. There is in all conscience much to be learned and laid to heart in fighting an anti-tuberculosis campaign: it is never too soon to begin that healthful if occasionally humiliating practice.

